# End stage renal disease in French Guiana (data from R.E.I.N registry): South American or French?

**DOI:** 10.1186/s12882-017-0614-6

**Published:** 2017-06-30

**Authors:** Dévi Rita Rochemont, Mohamed Meddeb, Raoul Roura, Cécile Couchoud, Mathieu Nacher, Célia Basurko

**Affiliations:** 1 Centre d’Investigation Clinique Epidémiologie Clinique Antilles Guyane CIC INSERM 1424, Centre hospitalier Andrée Rosemon, Rue des flamboyants BP 6006, 97306 Cayenne, French Guiana; 2 KAPA santé, Clinique Véronique, 1453 rte Baduel, 97300 Cayenne, French Guiana; 3 Association Traitement de l’Insuffisance Rénale en Guyane (ATIRG), Centre hospitalier Andrée Rosemon, 1361 rte Baduel, 97300 Cayenne, French Guiana; 4 Biomedecine Agency, La plaine-Saint Denis France, 1 avenue du Stade de, 93212 Saint-Denis La Plaine, France; 5 EA3593, UFR Médecine - Université des Antilles et de la Guyane, Cayenne, French Guiana

**Keywords:** Co-morbidities, Epidemiology, French Guiana, Incidence, Prevalence, End stage renal failure

## Abstract

**Background:**

End-Stage renal disease (ESRD) causes considerable morbidity and mortality, and significantly alters patients’ quality of life. There are very few published data on this problem in the French Overseas territories. The development of a registry on end stage renal disease in French Guiana in 2011 allowed to describe the magnitude of this problem in the region for the first time.

**Methods:**

Using data from the French Renal Epidemiology and Information Network registry (R.E.I.N). Descriptive statistics on quantitative and qualitative variables in the registry were performed on prevalent cases and incident cases in 2011, 2012 and 2013.

**Results:**

French Guiana has one of the highest ESRD prevalence and incidence in France. The two main causes of ESRD were hypertensive and diabetic nephropathies. The French Guianese population had a different demographic profile (younger, more women, more migrants) than in mainland France. Most patients had at least one comorbidity, predominantly (95.3%) hypertension. In French Guiana dialysis was initiated in emergency for 71.3% of patients versus 33% in France (*p* < 0.001).

**Conclusion:**

These first results give important public health information: i) End stage renal disease has a very high prevalence relative to mainland France ii) Patients have a different demographic profile and enter care late in the course of their renal disease. These data are closer to what is observed in the Caribbean or in Latin America than in Mainland France.

## Background

The irreversible alteration of renal function leads to chronic renal failure which may reach a end stage requiring dialysis for the patient to survive. It is estimated that nearly 1 in 100 persons is suffering from renal failure, among whom 0.13% have end stage renal disease (ESRD) [1]. In France, 37,430 persons underwent dialysis in 2010 and 29,841 received a kidney transplant.

ESRD is both a consequence and an aggravating factor of a number of cardiovascular and metabolic pathologies. It is associated with a considerable increase in morbidity and mortality, and significantly alters the patients’ quality of life [2]. In Mainland France and in Latin America, the 2 main reported causes of renal failure were, for over half of patients, diabetes mellitus and high blood pressure [3–5]. The increase of the incidence of these 2 pathologies raises the concern that the prevalence of chronic renal failure will increase in years to come.

In French Guiana, a French territory in South America between Brazil and Surinam, diabetes mellitus, which is probably underestimated, has increased by 57% between 2004 and 2007 [6]. The prevalence of high blood pressure in the French Caribbean and in French Guiana has been estimated at 18,9% for women, versus 9,3% in mainland France [7]. The frequency of stroke, the first cause of death in French Guiana, also reflects the problem represented by these pathologies in the Caribbean and in French Guiana. Mortality from stroke was 77.9% higher among men and 22.5% among women when compared to the national average [8].

There are few available data on renal failure in the French overseas territories of America. According to the literature, one in every three diabetic patients develops renal failure after 10 years of diabetes mellitus (6% having end stage renal disease) [9]. In addition French Guiana still has an important burden of infectious diseases notably HIV (Human Immunodeficiency Virus), which in itself, or through nephrotoxic treatments, can cause renal failure [10, 11]. French Guiana is also marked by socioeconomic differences which can lead to health inequalities regarding access to care. Thus, given the prevalence of metabolic and cardiovascular problems in French Guiana, it is important to better understand the profile of patients with ESRD in order to slow down or prevent the occurrence of this complication. The objective of the present study was to describe the epidemiology of ESRD in French Guiana and compare it to mainland France.

French Guiana has very specific geographic and demographic characteristics. With its 83,534 km^2^ French Guiana is the largest French overseas territory in the Americas representing 15% of the area of Mainland France, on par with Portugal, England or South Carolina. Population density is very low at 2.9 inhabitants/per km^2^, with marked heterogeneity, most of the population living along the coastline and the remaining living in small villages scattered in the interior. French Guiana has a very high population growth, 5 times that of mainland France, a growth rate which is the highest in Latin America, similar to that of Guatemala [12]. The population is thus very young, with 43% of the population aged younger than 20 years. The socioeconomic conditions of this French territory are 43% lower than in Mainland France [13]. In 2013, the unemployment rate was 32.8% [14]. However, despite these figures, French Guiana still has the highest GDP per capita in Latin America. Regarding access to care, some medical or surgical specialties are missing on the territory (Thoracic surgery, Cardiac Surgery, Burn wards, medically assisted procreation…). French Guianese patients are thus often evacuated to mainland France or Martinique for specialized procedures. Whereas patients living along the coast have similar average durations for access to care than in Mainland France, delays in accessing care are an issue for persons living in the interior who require lengthy helicopter transfers [15].

## Methods

### Population

The study was conducted using data from the cohort of patients included in the REIN registry for end stage renal disease between Jan 1st 2011 and December 31st 2013 in French Guiana. The study population corresponded to patients receiving treatment for ESRD by either dialysis or renal transplantation followed in one of the five centers treating such patients in French Guiana.

Between 2011 and 2013 a very low proportion of patients with end stage renal disease actually had renal biopsy: 13.5% (10.86% in 2011, 13% in 2012, and 16.57% in 2013). However, the diagnosis was performed by nephrologists after a complete but non invasive evaluation (biology on blood and urine, kidney and abdominal ultrasounds, abdominal scanner, and sometimes angiography.)

The R.E.I.N. network was developed by the Agence de Biomedecine (a public structure involved in 4 activities: organ, tissue, or cell transplants, procreation, embryology and human genetics). R.E.I.N uses 2 tools, Diadem for dialysed patients and Cristal for transplanted patients.

The cohort is approved by the French Regulatory authorities CNIL (Commission Nationale de l’Informatique et des Libertés, authorisation n° 903,188) and the CCTIRS (Comité Consultative sur le traitement de l’Information en matière de Recherche dans le domaine de la Santé, authorisation n°03.149)

### Registry data

The REIN (Réseau Epidémiologie et Information en Néphrologie) registry aims to describe the incidence and prevalence of end stage renal disease. It describes treatments, population characteristics, patient survival, and access to renal transplant. The registry aims to be exhaustive for patients with end stage renal disease. Research assistants compile clinical and para clinical data from medical records at the three sites dealing with patients with end stage renal disease in French Guiana. The registry includes mainland France and the overseas French territories which allows identifying epidemiologic differences and benchmarking.

The information collected upon inclusion included data on patient care structures and patient data including socio-demographic, initial renal disease, clinical state and the treatment modalities data. The patients were followed by the medical staff who prospectively collected events occurring during treatment, notably dialysis modality changes, transfer between structures, weaning, renal transplant attempts, and death.

Descriptive statistics on quantitative and qualitative variables were performed on prevalent and incident cases in 2011, 2012 and 2013. Pearson’s chi2 and Student’s t-test were used to compare groups. Comparisons between French Guiana, Mainland France and other French Territories was performed with age and sex-standardized rates using the direct standardization method (reference population France) to avoid distortions due to the different population age and sex structures. Data analysis was performed using STATA 11.1 (STATA Corp, College Station, Texas).

## Results

From 2011 to 2013, 309 patients were included in the registry. Among these patients, there were 99 incidents cases of ESRD (respectively 32, 28, 39 in 2011, 2012 and 2013) and 3 persons returning from a renal transplant in 2011. Seventy-two patients were died (respectively 22, 23, 27 in 2011, 2012 and 2013), ten patients were weaned, eighteen received renal transplant and thirty-one patients were transferred to another French territory or lost to follow-up.

**Fig. 1:**
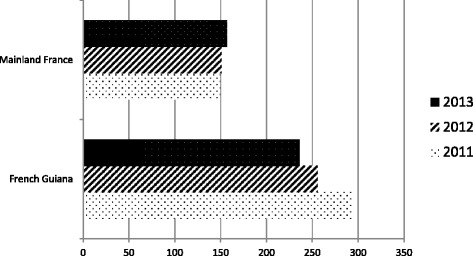
Incidence of treated ESRD on December 31 of each year (standardized rates per million populations) in French Guiana and Mainland France [40]

**Fig. 2:**
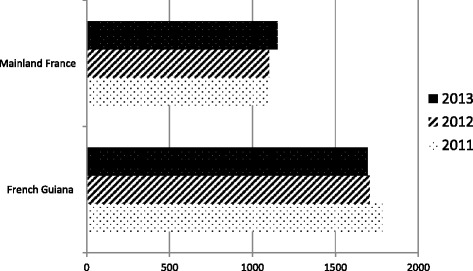
Prevalence of treated ESRD on December 31 of each year (standardized rates per million populations) in French Guiana and Mainland France [40]

After standardization, French Guiana was one of the three French territories with the highest ESRD prevalence and incidence. In 2011, ESRD incidence was 294 patients per million inhabitants in French Guiana, after the Reunion island (incidence of 412 per million inhabitants). In 2012 and 2013 French Guiana was still part of the first three French territories with the highest incidence (Fig. 1). The global ESRD prevalence was 1553 per million inhabitants in 2011, also behind the Reunion island (2792 patients per million inhabitants). In 2012 and 2013, French Guiana was still the second French territory with the highest prevalence of dialysis-treated ESRD (Fig. 2).

Mean age of patients was 51 years at dialysis onset (± 15.45). Dialyzed patients in French Guiana wereyounger than 65 years at dialysis treatment onset in 80.7% of cases, versus 38.4% of patients in mainland France over the three years, *p* < 0, 0001 (Table 1).

**Table 1:** Comparisons between French Guiana and Mainland France

	French Guiana	Mainland France	*P*
Patients <65 years old % (n)	80.7 (185)	38.4 (40889)	≤0.001
Sex ratio	1.3 (185)	1.5 (40889)	
Dialysis emergency % (n)	71.3 (99)	33 (9115)	≤0.001
Dialysis in an advanced medical center % (n)	68 (185)	59.3 (37032)	0.016
Initial vascular access with catheter % (n)	84 (185)	57 (40940)	≤0.001
Arterioveinous fistula vascular access % (n)	81 (185)	78.3 (34743)	0.374

The sex ratio of dialyzed patients was 1.3 men/woman whereas it was 1.5 in mainland France. For incident cases, in 2011, French Guiana was the only territory with a female biased sex ratio (0.9 men per woman).

Half of dialyzed patients in French Guiana (50%) were not born in France. The two other most represented countries were Suriname (17.57%) and Haïti (16.55%).

Of all patients included in the registry, the two most frequent underlying pathologies causing the nephropathies in patients with ESRD were high blood pressure (40.47%) and diabetes mellitus (28.95%). The main nephropathies of dialysis patients is presented for each year in Table 2 for French Guiana and for other French territories.

**Table 2:** Initial nephropathies among prevalent ESRD cases in French Guiana and France (on December 31th of each given year) [40]

Initial nephropathies	French Guiana *n* (%)			France *n* (%)			*p*-value		
	2011	2012	2013	2011	2012	2013	2011	2012	2013
Nephroangiosclerosis with renal failure (hypertensive nephropathy)	88 (47.8)	85 (47.75)	82 (43.16)	8718 (22.3)	9301 (22.7)	9725 (22.9)	<0.0000	<0.0000	<0.0000
Mesangial diffuse or nodular diabetic nephropathy	42 (22.8)	38 (21.35)	49 (25.79)	8181 (20.9)	8737 (21.3)	9152 (21.5)	0.76	0.99	0.47

**Fig. 3:**
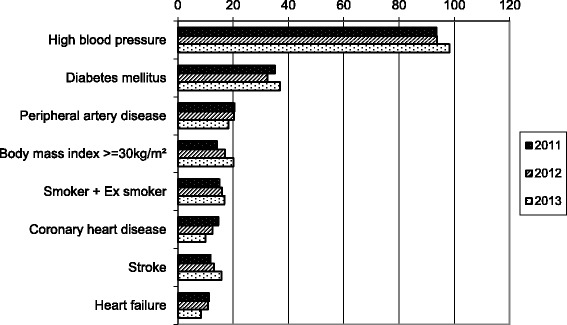
Principal co-morbidities of ESRD patients (on December 31 of each given year) in French Guiana

Overall, 91.9% had at least one co-morbidity or risk factor. High blood pressure was present in 95% of patients. Fig. 3 represents the 8 principal co-morbidities, which were present in more than 10% of patients.

Most patients had discovered ESRD at the onset of dialysis, and 65.1% ofpatients had required emergency dialysis. Among incident patients, 71.3% had initially emergency dialysis versus 33% in mainland France (*p* < 0.0000), (Table 1).

Concerning the modalities of dialysis in the study, 68% of patients were dialyzed in a medically advanced center (Mainland France: 59.3%, *p* < 0.016) whereas 33% were treated in an auto-dialysis center. Most patients were dialyzed through an arterioveinous fistula (81% in French Guiana, 78.3% in mainland France, *p* < 0.374). However, the first dialysis mostly used a catheter in French Guiana, 83.9% a higher proportion than in Mainland France (57%), *p* < 0.001, (Table 1). The mean weekly number of dialysis sessions was three and the mean duration per session was 4 h, as reported in Mainland France.

### Transplanted and deceased patients

From 2011 to 2013, eighteen patients received renal transplant, their mean age was 46.9 ± 12.9 years.

Seventy two patients died during this period, with a mean age at death of 65.3 ± 13.9 years. Most patients died at the hospital or in a private clinic (66.85%), the rest died at home (26.44%) or at the dialysis unit (5.47%). The two most frequent causes of death were cerebrovascular and cardiovascular disease.

## Discussion

Because of its location in the Amazon basin, French Guiana is more notorious for its infectious diseases than for its chronic diseases. However, the present results show that it is one the French territories that is most affected by ESRD. According to the cohort data, the epidemiologic profile of patients with ESRD was different from patients in mainland France. These differences were demographic (younger population, more women, half were migrants) but also regarding the initiation of care (mostly in emergency, using a catheter for the first dialyses).

The profile of patients dialysed in French Guiana resembled more what is observed in the Caribbean, or in Latin America, than in mainland France. In these regions, the mean age at the time of diagnosis of ESRD ranged from 47 years (in Jamaica) to 57 years (British Virgin Islands) [3, 4, 16]. The feminization of this pathology was observed in incident cases in French Guiana and in the Reunion Island [17]. This is presumably linked to the fact that, in these territories, diabetes mellitus affects more women than men, in contrast with mainland France.

In the Caribbean and in Latin America, diabetes mellitus and high blood pressure are the principal initial causes of nephropathy. In Latin America, half of the patients with high blood pressure at the time of diagnosis ESRD were not aware of having hypertension [4], underscoring the problem of early diagnosis of these causes of renal disease. In French Guiana, almost all patients in the registry had high blood pressure and nearly half had a hypertensive nephropathy, which is over the double of what is observed in mainland France. Moreover, African ancestry has been shown to be related to increased prevalence of hypertension and a greater fragility of target organs to hypertension when compared with Caucasians [18–20]. Regarding diabetes mellitus, the second comorbidity in our cohort, it has been shown that there are more treated cases of diabetes mellitus in the French overseas territories of the Americas than in mainland France, but that the results in term of glycemic control were not as good as in mainland France [21, 22]. Several studies have also shown that afro-caribbean and indo-asian ancestry were associated with a greater prevalence of diabetes mellitus and thus was associated with a greater risk of chronic renal failure [22–24]. Afro-Caribbean populations, largely represented in French Guiana, are thus more likely to develop hypertension and diabetes mellitus, the two main causes of chronic renal failure [25–27].

There are however other specificities to French Guiana than its ethnic mix. The emergency context of initial care of ESRD had been observed in mainland France for several years, however in French Guiana, this delay before care was even greater Seven out of 10 patients had initiated dialysis in an emergency context in French Guiana versus three out of ten patients in mainland France. Several countries have emphasized that initial care in an emergency context was associated with complications, increased hospital stay, a reduction in autonomy and increased mortality in the first month of dialysis [28–30]. The present results raise the problem of early diagnosis and care of risk factors of ESRD, like in Latin America [3]. The early implementation of therapeutic recommendations for diabetes mellitus [31], and hypertension [31, 32], have shown to improve the progression of renal disease. The “practice guidelines for chronic kidney diseases” recommend a practical clinical action plan for each patient based on the stage of his disease [33]. Early detection by routinely controlling marquers of renal disease is important [33, 34] to reduce the proportion of patients with a late diagnosis of renal disease. Systematic screening for comorbidities such as high blood pressure or diabetes mellitus seem like effective strategies to delay or even prevent the need for dialysis. The lack of nephrologists and diabetologists in French Guiana could also explain the treatment delay. Indeed, a demographic study in France showed that regions with a low density of nephrologists were those where the proportion of renal failure patients was highest [35]. Patient care would also improve if first line health care professionals and specialists in dialysis centers were better coordinated [2, 30, 36–38] to avoid late referral, which is associated with increased mortality after the initiation of dialysis [33]. Overall, given the scarcity of nephrologists in French Guiana, organisational efforts are needed to implement coordinated multisectoral care of patients. Although, there were no data on socioeconomic level and access to rights and access to care, the progression of high blood pressure or diabetes mellitus towards complications may reflect delayed access to care for the more vulnerable segment of the population in French Guiana [15, 39]. Disentangling what results from disorganization and low numbers of health practitionner, from patient’s representations and health seeking behaviours and from true obstacles to care is an important research question that may guide future interventions to improve these preoccupying figures.

## Conclusion

In France, French Guiana is perceived as a hostile territory still struggling with numerous tropical diseases. However, the epidemiologic transition from infectious diseases to chronic diseases is now quite apparent in the local burden of disease. Whereas infectious diseases specialists are drawn to the challenges of this” tropical medicine hotspot”, the rising problem of chronic diseases fails to do so despite important needs and a lack of specialized professionals. The first results of the end stage renal disease registry in French Guiana give important public health informations: i) it shows the high prevalence of end stage renal disease in this territory ii) it shows the atypical profile of patients and the late access to care which are closer to Caribbean or south American patient characteristics. It would also be important to adapt public health measures to the local epidemiology and notably improve access to care and early management of the main causes of renal failure in French Guiana.

## Abbreviations

ATIRG: Association Traitement de l’Insuffisance Rénale en Guyane
CCTIRS: Comité Consultative sur le Traitement de l’Information en matière de Recherche dans le domaine de la Santé
CNIL: Commission Nationale de l’Informatique et des Libertés
ESRD: End-stage renal disease
HIV: Human Immunodeficiency Virus
R.E.I.N: Renal Epidemiology and Information Network registry
